# Pre-transplant Biomarkers of Immune Dysfunction Improve Risk Assessment of Post-transplant Mortality Compared With Conventional Clinical Risk Scores

**DOI:** 10.21203/rs.3.rs-2548184/v1

**Published:** 2023-02-06

**Authors:** J. Esli Medina-Morales, Guergana G. Panayotova, Duc T. Nguyen, Edward A. Graviss, Gagan S. Prakash, Jeffery A. Marsh, Sopio Simonishvili, Yash Shah, Tumininu Ayorinde, Yong Qin, Lianhua Jin, Theofano Zoumpou, Laurie J. Minze, Flavio Paterno, Arpit Amin, Grace Lee Riddle, R. Mark Ghobrial, James V. Guarrera, Keri E. Lunsford

**Affiliations:** Rutgers New Jersey Medical School; Rutgers New Jersey Medical School; Houston Methodist Research Institute; Houston Methodist Research Institute; Rutgers New Jersey Medical School; Rutgers New Jersey Medical School; Rutgers New Jersey Medical School; Rutgers New Jersey Medical School; Rutgers New Jersey Medical School; Rutgers New Jersey Medical School; Rutgers New Jersey Medical School; Rutgers New Jersey Medical School; Rutgers New Jersey Medical School; Rutgers New Jersey Medical School; Rutgers New Jersey Medical School; Rutgers New Jersey Medical School; Houston Methodist Hospital; Rutgers New Jersey Medical School; Rutgers New Jersey Medical School

**Keywords:** Liver Transplant, Cirrhosis, Pre-Transplant Risk Assessment, Liver Transplant Futility, Clinical Risk Score, Post-Transplant Mortality

## Abstract

**Introduction:**

There is a critical need to accurately stratify liver transplant (LT) candidates’ risk of post-LT mortality prior to LT to optimize patient selection and avoid futility. Here, we compare current *pre*-LT clinical risk scores with the recently developed Liver Immune Frailty Index (LIFI) for prediction of *post*-LT mortality. LIFI measures immune dysregulation based on pre-LT plasma HCV IgG, MMP3 and Fractalkine. LIFI accurately predicts post-LT mortality, with LIFI-low corresponding to 1.4% 1-year post-LT mortality compared with 58.3% for LIFI-high (C-statistic=0.85).

**Methods:**

LIFI was compared to MELD, MELD-Na, MELD 3.0, D-MELD, MELD-GRAIL, MELD-GRAIL-Na, UCLA-FRS, BAR, SOFT, P-SOFT, and LDRI scores on 289 listed patients (T_0_). Survival, hazard of early post-LT death, and discrimination power (C-statistic) were assessed (Stata v17).

**Results:**

LIFI showed superior discrimination (highest C-statistic) of post-LT mortality when compared to all other risk scores, irrespective of biologic MELD. On univariate analysis, the LIFI showed a significant correlation with mortality at 3- and 6-months, as well as 1-, 3-, and 5-years. *No other pre-LT scoring system significantly correlated with post-LT mortality*. On bivariate adjusted analysis, African American race and pre-LT cardiovascular disease were significantly associated with early- and long-term post-LT mortality (p<0.05). Patients who died within 1-yr following LT had a significantly higher incidence of infections, including 30-day and 90-day incidence of any infection, pneumonia, abdominal infections, and UTI (p<0.05).

**Conclusions:**

LIFI, which measures pre-LT biomarkers of immune dysfunction, more accurately predicts risk of post-LT futility compared with current clinical predictive models. Pre-LT assessment of immune dysregulation may be critical in predicting mortality after LT and may optimize selection of candidates with lowest risk of futile outcomes.

## 1 Introduction

Liver transplant (LT) is the only curative treatment for end-stage liver disease (ESLD); however, due to the current shortage of donor organs, as many as one third of patients on the waiting list will expire awaiting LT (1). The current organ allocation model relies on the “Final Rule,” based on the principle of transplanting the “sickest first” while avoiding LT in patients at risk for futile outcomes (2, 3).To accomplish this, patients are risk-stratified by using the Model for End Stage Liver Disease (MELD) (4, 5). Despite its clinical utility in estimating risk of wait-list mortality, MELD fails to accurately predict the risk of death *following* LT (6). Additional scoring systems have been proposed to better risk-stratify patients.

MELD was initially developed to assess mortality in cirrhotic patients undergoing trans jugular intrahepatic portosystemic shunts (TIPS) (7). Subsequently, MELD has been validated to predict three-month waitlist mortality in candidates awaiting LT (5). MELD was later updated to include sodium, which is an independent risk factor of waitlist mortality, resulting in current use of MELD-Na as the pre-LT acuity score (8). Several variations of MELD have been proposed to increase sensitivity for severe disease, including the addition of variable such as female sex and albumin level to account for allocation inequities. To capture renal function more accurately in the cirrhotic patient, MELD 3.0 (9) and GFR assessment of liver disease (GRAIL) scores (MELD-GRAIL and MELD-GRAIL Na) were developed (10). Incorporating these additional components increases sensitivity for 90-day waitlist mortality, especially in decompensated cirrhotics and women (11). MELD variations were developed for assessment of *pre*-LT mortality, but application to predict *post*-transplant outcomes have been deficient.

Additional scores have been proposed specifically for pre-LT risk assessment of post-LT mortality. These commonly incorporate recipient co-morbidities in addition to donor factors. The D-MELD combines the donor’s age and the recipient’s preoperative calculated MELD score to predict survival and length of hospitalization (LOS) after LT (12). The Balance of Risk (BAR) score is a simplified score that includes recipient age, MELD, life-support requirement, and retransplant in addition to donor age and cold ischemia time (13). More complex scores, such as the Survival Outcomes Following liver Transplantation (SOFT) score, utilize 18 donor, recipient, and graft variables (14). A variation including only pre-procurement variables (P-SOFT) can risk stratify waitlisted patients (14). The liver donor risk index (LDRI) determines risk of post-liver transplant graft failure based on seven donor and graft characteristics (15). Finally, the UCLA Futility Risk Score (UCLA-FRS) further discriminates risk of post-LT futility in patients with MELD scores ≥ 40, using the age adjusted Charlson Comorbidity Index (CCI)(16), MELD, sepsis within 30 days, and cardiac risk (17).

Of these risk stratification tools, BAR and SOFT have the highest previously described discrimination for pre-LT prediction of post-LT mortality; however, none have a concordance (C-)statistic > 0.7 (8–15, 17, 18). In addition, many incorporate donor and intraoperative information, which limits their utility prior to donor selection (13, 14). Ultimately, recipient candidacy relies heavily on subjective clinical judgment and is prone to selection bias (19). Objective parameters to accurately stratify patients’ risk of post-LT death prior to LT are lacking. This, in conjunction with increasing recipient severity of illness at the time of transplant (17, 20–23), risks futile LT. There is a critical need to accurately stratify LT candidates’ risk of post-LT mortality prior to LT to optimize patient selection and avoid futility.

Transplant candidate immune dysfunction appears to be a critical component of overall patient severity of illness and susceptibility to complications. Cirrhosis ultimately leads to profound metabolic and immunologic dysfunction (24, 25), resulting in both physical and immunologic frailty (23). Despite a clear association with post-transplant outcomes, immune dysfunction is not currently utilized in pre-LT candidate assessment. We have recently introduced a novel pre-LT risk score, the Liver Immune Frailty Index (LIFI), based on pre-transplant biomarkers of immune dysfunction. Using weighted points assigned to pre-LT HCV-IgG status and plasma concentrations of Fractalkine and MMP3, LIFI stratifies patients into high-, moderate-, and low-risk of early post-LT mortality (death ≤ 1 year). On internal validation, LIFI predicts futility with C-statistic = 0.84, which exceeds predictive capacity of previously described models. Specifically, LIFI-high LT recipients had a 58.3% risk of 1-year mortality post-LT compared to 12.7% for LIFI-moderate and 1.4% for LIFI-low (23).

Here, we compare the discrimination power of LIFI for predicting 1-year and long-term post-LT mortality to currently utilized risk scores for LT candidate assessment in the same patient cohort. Outcomes are further stratified by recipient MELD score <30 vs ≥ 30, as recent studies show MELD > 30 is an independent risk factor for poor clinical outcomes following LT (26, 27)

## 2 Methods

### 2.1. Study cohort

Adult patients on the waitlist for liver transplant at Houston Methodist Hospital (HMH, January 1, 2013 – December 31, 2017) and University Hospital/Rutgers New Jersey Medical School (Rutgers NJMS, January 1, 2018 – December 31, 2021) were evaluated for inclusion. All protocols were approved by each institution’s respective institutional review board (IRB) and human subject research was conducted in accordance with the ethical principles of the Declaration of Helsinki (28).

**Figure 1:**
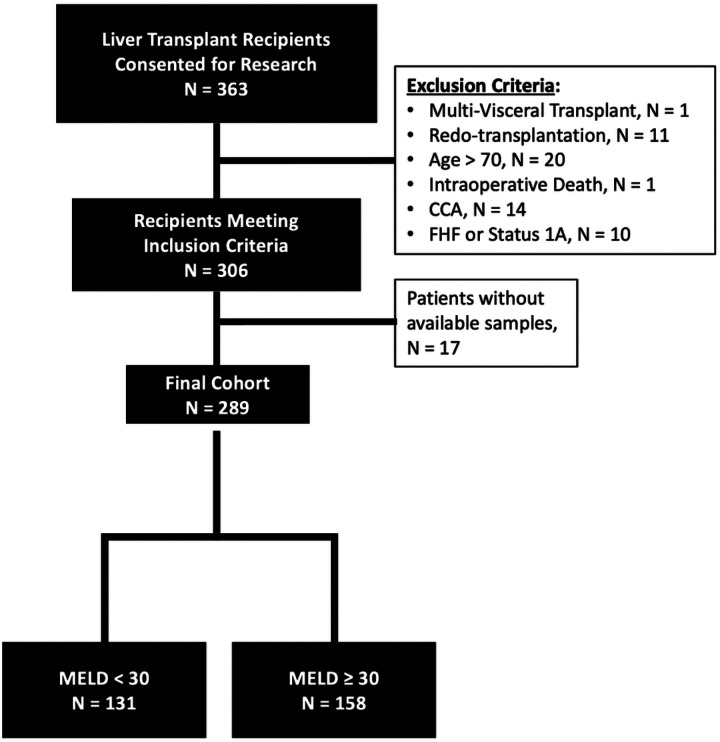
Flowchart diagram of inclusion and exclusion criteria.

Adult recipients (age ≥ 18 years old) who received a deceased donor whole LT with at least 12 months of follow-up were considered for analysis. Patients with a history of cholangiocarcinoma, fulminant hepatic failure, expiring during the transplant procedure, or receiving multi-visceral transplants other than liver-kidney were excluded. A total of 289 patients were included in the final analysis (Fig. 1).

### 2.2. Data collection

Pre-LT recipient demographic data (age, sex, race, weight, height, BMI, primary cause of end-stage liver disease [ESLD]) and comorbidities (liver cold ischemia time, prior abdominal surgery, chronic renal insufficiency [CRI], diabetes mellitus, cardiac comorbidities [prior MI, stent, valvular insufficiency, and arrythmia], peptic ulcer disease, chronic pulmonary disease, cancer, and clinical findings of portal hypertension [ascites, encephalopathy, variceal bleeding]) were documented. Pre-LT medical acuity was recorded, including MELD of MELD-Na, vasopressors, ventilator, dialysis, hospital length-of-stay (LOS), and intensive care unit (ICU) LOS. Pre-LT infections (defined as occurring 30 days prior to LT) were assessed, including pneumonia, peritonitis, sepsis, and urinary tract infection (UTI). Donor demographics include age, cause of death, terminal creatinine, deceased donor type (brain death vs. circulatory death), and location. Recipient laboratory data (albumin, sodium, creatinine, total bilirubin, AST, ALT, INR, hemoglobin, platelet count, and white blood cell count with differential) were obtained at the time of transplant. Both calculated biologic laboratory and list MELD scores were included, as reported to the United Network of Organ Sharing (UNOS). The MELD-Na was utilized for all patients following policy induction after January 2016 (29).

The primary outcome was patient survival, assessed at 3- and 6-months, as well as 1-, 3-, and 5-years post-LT. Secondary outcomes included post-LT complications, including death, hospital length of stay, ICU length of stay, presence of cardiac morbidity after LT, and infectious complications within 30 and 90-days after LT. Severe infection was defined as sepsis, pneumonia, or intra-abdominal infection within 90 days following LT.

### 2.3. Score model calculations

The LIFI score was calculated as previously described(23). LIFI was compared to currently accepted medical acuity scoring systems suggested for the liver transplant population. Specifically, the MELD, MELD-Na, MELD 3.0, D-MELD, MELD-Grail, Meld Grail-Na, UCLA-FRS, BAR, SOFT, P-SOFT, and LDRI (5, 8, 9, 11–15, 17). Formulas used to calculate each score and their distribution can be found in **Supplemental Tables 1**.

### 2.4. Statistics

Continuous variables are reported as median and interquartile range (IQR) and categorical variables as frequencies and proportions. Differences between groups were compared using the ANOVA or the Kruskal Wallis test for continuous variables and the Chi-square or Fisher’s exact tests for categorical variables, as appropriate. Bivariate analysis was used to evaluate patient characteristics, stratified by post-transplant mortality at 3- and 6-months, as well as 1-, 3-, and 5-years. Univariate Cox regression modeling was used to determine the hazard ratio for post-transplant mortality at 3- and 6-months, as well as 1-, 3-, and 5-years of individual risk scores and their components. The discrimination power of individual risk scores was determined by the C-statistic and compared with that of the LIFI score using the chi-square test. All analyses were performed on Stata version 17.0 (StataCorp LLC, College Station, TX, USA). A p-value of < 0.05 was considered statistically significant.

## 3 Results

### 3.1 Comparison of donor and recipient demographics.

A total of 289 patients receiving LTs between January 2013-December 2021 and were included in final analysis (Fig. 1). Overall patient demographics are shown in **Table 1**. LT recipient outcomes were compared for patient who survived > 1 year following LT versus those with death within 1 year (**Table 1**). Black race (p = 0.02), history of cardiovascular disease (p = 0.05), and intraabdominal infection within 30 days of LT (p = 0.01) were associated with 1-year post-LT mortality (**Table 1**). On bivariate analysis based on time to mortality, only pre-LT cardiac morbidity was significantly associated with early mortality at 6-months and 1-year. Long-term mortality (3- and 5-year) was associated with older age at LT (**Supplemental Table 2**).

### 3.2. MELD Score Correlates with Pre-LT Patient Severity of Illness but Fails to Discriminate Patient Mortality

Patients were next stratified into subgroups based on biologic MELD at LT (MELD ≥ 30, N = 158 and MELD <30, N = 131). Overall study population and subgroup patient characteristics are shown in Table 2. Compared to MELD ≥ 30, recipients with MELD > 30 were older (54.0 vs 58.3 years, p < 0.01), had higher BMI (29.8 vs 27.1 kg/m^2^, p< 0.001), less commonly had HCC (5.7% vs 45%, p< 0.001), and varied with regards to liver disease etiology (p < 0.01, Table 2). As expected, chronic renal insufficiency (CRI) was more common in MELD ≥ 30 (31.2%vs 17.6%, p = 0.01). The higher MELD cohort also had a significantly higher incidence of peptic ulcer disease (PUD), encephalopathy at LT, pre-LT infections, and ICU-level care. Finally, recipients with MELD ≥ 30 received grafts from younger, locally located, and donation after brain death (DBD) donors, (p < 0.05, Table 2).

Following transplant, recipients with MELD ≥ 30 experienced more frequent blood transfusions (82.2% vs 63.5%, p = 0.01) and had longer median overall LOS (21 vs 12 days, p< 0.001) as well as ICU LOS (14 vs 5 days, p< 0.001). The incidence of post-transplant infections, including any positive cultures, documentation of sepsis, pneumonia, UTI, and intra-abdominal infection within 30- and 90-days were also significantly higher in MELD ≥ 30 vs MELD <30, p < 0.05 for all. Despite the overwhelming differences in pre-and early post-transplant morbidity, there was no statistically significant difference in patient status (alive vs dead, p = 0.95), nor patient survival days (p = 0.59) or survival months (p = 0.59) between groups (Table 3).

### 3.3. Clinical Risk Scores Correlate with Medical Acuity and MELD but Fail to Predict Mortality

Previously described risk scores were calculated for all patients and compared between the MELD < 30 and the MELD ≥ 30 cohort (**Supplemental Table 1**). As expected, based on calculation parameters, the MELD, MELD-Na, MELD 3.0, D-MELD, MELD-GRAIL, MELD-GRAIL Na, UCLA FRS, BAR, SOFT and P-SOFT were significantly higher in the high-MELD group (Table 4). The LDRI was the only score with significantly lower median values in the MELD ≥ 30 recipients (1.2 vs 1.3, p = 0.02), highlighting the potential bias in allocation and liver offer acceptance while matching presumed donor illness with recipient medical acuity. We next assessed the LIFI score in high and low MELD subgroups. There was no difference in overall LIFI score between MELD subgroups; however, when comparing LIFI tertiles of risk, LIFI-moderate and LIFI-high were significantly more frequent in the MELD ≥ 30 group (66.4%) compared to MELD < 30 (Table 4). These results highlight that aggregate LIFI score assesses parameters of recipient risk which are distinct from those evaluated by standard clinical risk scores.

### 3.3. LIFI has superior discrimination compared to all other clinical risk scores in both early and long-term post-LT mortality

On univariable Cox proportional hazard analysis, the LIFI score was the *only* pre-transplant risk score with a significant correlation with early post-LT mortality at 3- (HR 1.04 [1.01, 1.07] p = 0.003) and 6-months (HR 1.04 [1.03, 1.06] p < 0.001). This was observed for overall score, individual components, as well as when evaluated by LIFI tertiles of risk (Table 5). Similarly, LIFI was also the only score to significantly predict long-term mortality at 1- (HR 1.05 [1.04, 1.07] p < 0.001), 3- (HR 1.05 [1.04, 1.06] p < 0.001), and 5-years post-LT (1.04 [1.03, 1.06] p< 0.001; respectively) (Table 5). Finally clinical risk scores were assessed for discrimination power of 1-year mortality, using the C-statistic. LIFI showed superior discrimination (highest C-statistic) of post-LT mortality when compared to all other risk scores, irrespective of biologic MELD. This was especially pronounced in patients with MELD < 30 (C-statistics 0.92) (Table 5).

## Discussion

The “Final Rule” dictates allocation of livers in order of decreasing medical urgency (i.e. sickest first) while avoiding futile liver transplantation (LT) (2, 3). The current allocation system in the US stratifies pre-transplant illness severity based on the MELD score, which predicts three month waitlist mortality with C-statistic of 0.78–0.87 (4, 5). MELD, however, is a poor predictor of post-transplant mortality (C-statistic = 0.44–0.53) (6, 30). Other previously described pre-LT clinical scoring models either do not correlate with outcomes or require knowledge of donor and intraoperative information for calculation, which are not known prior to donor allocation. Multiple models have been described, all with c-statistic ≤ 0.7 for prediction of post-LT outcomes. Rising organ demand in conjunction with increasing recipient severity of illness necessitates a reliable method to risk-stratify critically ill patients based on their pre-LT severity of illness to avoid futile liver transplantation.

We have previously described the Liver Immune Frailty Index (LIFI), a biomarker panel based on HCV IgG status and plasma levels of MMP-3 and Fractalkine, which quantifies *pre-LT immune* dysfunction (a.k.a., immune frailty) and predicts risk of post-LT futility (23). Whether this model outperforms other conventional clinical scoring models was not known. Here, we find that LIFI significantly correlates with liver transplant recipient mortality at 3- and 6-months, as well as at 1-, 3-, and 5-years post-transplant. In addition, LIFI shows superior discrimination (highest C-statistic) of 1 -year post-LT mortality compared to all other risk scores, regardless of biologic MELD.

MELD and other conventional clinical scoring tools rely on laboratory values as surrogates for illness severity (8–15, 17, 18); however, these disregard the immunological status of patients at the time of LT. Infection is the leading cause of mortality within the first year following liver transplant, and ongoing infection risk likely results from persistent immune dysfunction following liver transplant. Pretransplant immune dysfunction in cirrhosis arises from the physiologic and metabolic alterations associated with progressive liver decompensation. This leads to cirrhosis associated immune dysfunction (CAID), which is characterized by deficiency in both innate and adaptive immunity, resulting from chronic immune system stimulation of liver injury, pathogenic infections, and gut-derived antigens (1). Chronic immune stimulation and exhaustion of metabolic substrates ultimately induces an inappropriate compensatory anti-inflammatory response. In the setting of severe decompensation, cirrhotic patients exhibit impaired immune response to bacterial challenge, which can result in severe systemic infection, multi-organ failure, and short-term mortality (31, 32) In its most severe form, *immune frailty*, pre-transplant immune dysfunction likely persists post-transplant and is exacerbated by immunosuppressive medications.

Prior clinical scoring systems have failed to capture the risk imparted by this severe state of ongoing immune dysfunction. This is a critical flaw that limits their clinical utility, as, infection is the leading cause of early post-transplant mortality. Of previously described models, three have shown the best sensitivity and specificity for predicting post-LT outcomes. These include the SOFT, BAR, and the UCLA-FRS scores (13, 14, 17). The SOFT score (14, 18) and BAR score (6) were created from patient-level data from the UNOS database, which despite its statistical power, lacks granularity to capture variables of immune dysfunction and infection risk. In addition, both scores require knowledge of donor characteristics and fail to consider recipient comorbidities, which are critical risk factors considered before waitlist placement (33). For that purpose, the UCLA-FRS score was created. This index was created through retrospective assessment of single center data, albeit at the center with the largest longitudinal liver transplant experience in the US. The single-center study design improved granularity, allowing inclusion of comorbidity history through adjusted Charlson comorbidity index (CCI) and cardiac risk. In addition, it is the only score to include any markers of pretransplant immune dysfunction; as, pre-transplant sepsis within 30 days of transplant likely reflects immune dysregulation (17). The original derivation of the UCLA-FRS, however, included only patients with MELD ≥ 40. Follow-up validation studies have demonstrated subpar performance in patients with lower pre-transplant severity of illness (threshold of MELD at 30, c-statistic of 0.65) (6). Thus, an objective and replicable system which considers immune dysfunction is necessary to improve pre-transplant risk-stratification of post-LT mortality.

Our recently described LIFI score stratifies patients into high-, moderate-, and low-risk of post-LT mortality. Patients with high-LIFI had a 1-year post-LT mortality of 58.3% compared to 1.4% in low-LIFI recipients (23). With a c-statistic of 0.84 in our cohort, LIFI is emerging as a potentially superior tool to support and guide clinical decision-making to avoid futile outcomes in high-risk LT recipients. Of note, LIFI offers superior discrimination of patient risk of mortality regardless of pre-LT MELD. Other clinical models have failed to accurately forecast outcomes in the low MELD cohort. Patients receiving liver transplant at lower MELD scores commonly have MELD exceptions, allowing their waitlist prioritization, with exception most commonly being granted for hepatocellular carcinoma. This may suggest that LIFI is able to discriminate not only the risk of mortality due to immune dysfunction relating to sepsis, but LIFI may also correlate with the risk of mortality related to recurrent cancer. Given that immune dysregulation allows tumor cells to escape immune surveillance, persistent immune dysfunction following liver transplant may increase a recipient’s risk of developing de novo or recurrent disease. Additional studies are necessary to delineate this relationship further.

There are several limitations to our findings. First, the LIFI was internally validated using granular patient-level data and immunologic assessment from patients at only two transplant centers. In addition, the LIFI was calculated via boot-strapping techniques, which does not consider changes in patient population while modeling prediction (34, 35). A large multi-center validation cohort is necessary to verify the model. In addition, due to the use of a limited patient cohort, we were not able to perform a multivariate prediction of 1-year post-LT mortality using components of the different pre-transplant scoring models given the small numbers of events at 1 year. LIFI includes HCV IgG status in its calculation. HCV likely figures more heavily into the risk score given that the discovery cohort used spanned the era of introduction of direct-acting antiviral medication when transplant was more common for HCV. As patient demographics change, we may see an era effect in significantly associated immune biomarkers, resulting in LIFI score adjustment. Finally, there is potential for selection bias given that certain subgroups were excluded during creation of LIFI, including re-transplant recipients, patients of advance age, and patients with fulminant hepatic failure. Additional analysis is necessary to evaluate LIFI in these cohorts.

In conclusion, LIFI predicts patient survival and is the only score to significantly correlate with mortality in both high and low MELD recipients. Pre-LT assessment of immune dysregulation may be critical in predicting mortality after LT and may optimize selection of candidates with lowest risk of futile outcomes.

## Figures and Tables

**Table 2:** Study population, stratified by MELD

	Total N = 289	Lab MELD <30 N = 131	Lab MELD ≥30 N = 158	p-value
**Recipient Demographics**				
Age at OLT, median (IQR)	56.6 (48.2, 62.5)	58.3 (52.5, 63.4)	54.0 (45.6, 60.7)	<0.001
Male gender	197 (68.2)	92 (70.2)	105 (66.5)	0.49
Recipient race				
Caucasian	177 (61.2)	82 (62.6)	95 (60.1)	0.39
African American	33 (11.4)	17 (13.0)	16 (10.1)	
Hispanic	62 (21.5)	23 (17.6)	39 (24.7)	
Asian	8 (2.8)	3 (2.3)	5 (3.2)	
Other	9 (3.1)	6 (4.6)	3 (1.9)	
Recipient race - Black	33 (11.4)	17 (13.0)	16 (10.1)	0.45
SKLT	35 (12.1)	13 (9.9)	22 (13.9)	0.30
BMI (kg/m2), median (IQR)	28.5 (24.8, 32.6)	27.1 (24.1, 30.5)	29.8 (25.5, 34.4)	<0.001
Cause of ESLD Code				
HCV	80 (27.7)	60 (45.8)	20 (12.7)	<0.001
EtOH	110 (38.1)	28 (21.4)	82 (51.9)	
NASH/Cryptogenic	57 (19.7)	22 (16.8)	35 (22.2)	
AIH	12 (4.2)	2 (1.5)	10 (6.3)	
PBC	2 (0.7)	0 (0.0)	2 (1.3)	
PSC	5 (1.7)	2 (1.5)	3 (1.9)	
HCC	2 (0.7)	1 (0.8)	1 (0.6)	
PCLKD	10 (3.5)	10 (7.6)	0 (0.0)	
**Recipient Demographics**				
HBV	6 (2.1)	3 (2.3)	3 (1.9)	
FHF	2 (0.7)	1 (0.8)	1 (0.6)	
Other	3 (1.0)	2 (1.5)	1 (0.6)	
CRI	72 (25.0)	23 (17.6)	49 (31.2)	0.01
Diabetes	97 (33.6)	46 (35.1)	51 (32.3)	0.61
CAD	22 (7.6)	8 (2.9)	2 (11.8)	0.05
PVD	6 (2.1)	1 (0.8)	5 (3.2)	0.15
COPD	19 (6.6)	6 (4.6)	13 (8.2)	0.21
Connective Tissue Disease	6 (2.1)	1 (0.8)	5 (3.2)	0.15
PUD	23 (8.0)	5 (3.8)	18 (11.4)	0.02
**Pre-transplant Labs, median (IQR)**				
T. bilirubin (mg/dL)	8.7 (1.5, 22.9)	1.4 (0.7, 3.5)	19.8 (10.3, 29.9)	<0.001
INR	2.0 (1.4, 2.5)	1.3 (1.1, 1.7)	2.4 (2.0, 2.9)	<0.001
BUN (mg/dL)	28.4 (22.0, 42.0)	26.2 (19.2, 32.5)	32.1 (24.7, 53.6)	<0.001
Creatinine (mg/dL)	1.1 (0.8, 1.8)	0.9 (0.7, 1.3)	1.4 (1.0, 2.3)	<0.001
Albumin (g/L)	3.1 (2.7, 3.6)	3.3 (2.7, 3.7)	3.0 (2.7, 3.6)	0.31
Sodium (mEq/L)	139.0 (136.0, 141.0)	139.0 (136.0, 141.0)	139.0 (136.0, 141.0)	0.43
**Pre-transplant Medical Acuity**				
Pressors	62 (21.5)	7 (5.3)	55 (34.8)	<0.001
Vent	80 (27.7)	8 (6.1)	72 (45.6)	<0.001
**Recipient Demographics**				
LOS (days), median (IQR)	7.0 (1.0, 17.0)	1.0 (0.0, 1.0)	15.0 (9.0, 23.0)	<0.001
ICU LOS (days), median (IQR)	0.0 (0.0, 7.0)	0.0 (0.0, 0.0)	6.0 (0.0, 16.0)	<0.001
RRT	126 (43.6)	20 (15.3)	106 (67.1)	<0.001
**Pre-transplant Infections (30 days prior to LT)**				
Any Positive Cultures	104 (36.0)	27 (20.6)	77 (48.7)	<0.001
Sepsis	32 (11.1)	3 (2.3)	29 (18.4)	<0.001
Pneumonia	39 (13.5)	7 (5.3)	32 (20.3)	<0.001
UTI	57 (19.7)	18 (13.7)	39 (24.7)	0.02
Intraabdominal Infection	21 (7.3)	4 (3.1)	17 (10.8)	0.01
**Donor Characteristics**				
Donor Age	31.0 (24.0, 45.0)	36.0 (26.0, 50.0)	29.5 (22.0, 39.0)	<0.001
Donor Location				<0.001
Local	163 (56.4)	95 (72.5)	68 (43.0)	
Regional	114 (39.4)	32 (24.4)	82 (51.9)	
National	12 (4.2)	4 (3.1)	8 (5.1)	
Donor Type				0.03
DBD	275 (95.2)	120 (91.6)	155 (98.1)	
DCD	13 (4.5)	10 (7.6)	3 (1.9)	

IQR, interquartile range mean; OLT, orthotopic liver transplantation; SKLT, Simultaneous Liver Kidney Transplant; BMI, body mass index; ESLD, end-stage liver disease; HCV, hepatitis C; EtOH, alcohol abuse disorder; NASH, non-alcoholic steatohepatitis; CAD, coronary artery disease; PVD, peripheral vascular disease; COPD, chronic pulmonary disease; PUD, peptic ulcer disease; INR, international normalized ratio; bUn, blood urea nitrogen; CRI, chronic renal insufficiency; LOS, length of hospitalization; ICU, intensive care unit; UTI, urinary tract infection; DBD, donation after brain death; DCD, donation after cardiac death.

**Table 3:** Post-transplant mortality and morbidity stratified by MELD score

	Total	Lab MELD <30	Lab MELD ≥ 30	
	(N = 289)	(N = 131)	(N = 158)	p-value
**Post-transplant mortality**				
Patient Status				0.95
Alive	240 (83.0)	109 (83.2)	131 (82.9)	
Dead	49 (17.0)	22 (16.8)	27 (17.1)	
Survival Days, median (IQR)	1458.0 (799.0, 1895.0)	1458.0 (1122.0, 1860.0)	1456.5 (705.0, 2071.0)	0.59
Survival Months, median (IQR)	47.9 (26.3, 62.3)	47.9 (36.9, 61.2)	47.9 (23.2, 68.1)	0.59
Recipient COD				0.054
Unknown/Other	5 (9.8)	2 (8.7)	3 (10.7)	
Renal Failure	1 (2.0)	0 (0.0)	1 (3.6)	
Sepsis/Infection	19 (37.3)	5 (21.7)	14 (50.0)	
Cardiac	4 (7.8)	1 (4.3)	3 (10.7)	
Graft Failure	6 (11.8)	2 (8.7)	4 (14.3)	
Malignancy/Other	2 (3.9)	2 (8.7)	0 (0.0)	
Recurrent HCC/CCA	7 (13.7)	6 (26.1)	1 (3.6)	
Pulmonary	1 (2.0)	0 (0.0)	1 (3.6)	
Neurologic	3 (5.9)	3 (13.0)	0 (0.0)	
Suicide	1 (2.0)	1 (4.3)	0 (0.0)	
GVHD	1 (2.0)	0 (0.0)	1 (3.6)	
PTLD	1 (2.0)	1 (4.3)	0 (0.0)	
Patient Survival 90d				0.46
Alive	286 (99.0)	129 (98.5)	157 (99.4)	
Dead	3 (1.0)	2 (1.5)	1 (0.6)	
**Post-transplant mortality**				
Patient Survival 1 yr				0.98
Alive	269 (93.1)	122 (93.1)	147 (93.0)	
Dead	20 (6.9)	9 (6.9)	11 (7.0)	
LOS (days), median (IQR)	17.0 (11.0, 28.0)	12.0 (9.0, 20.0)	21.0 (15.0, 34.0)	<0.001
ICU LOS (Days), median (IQR)	9.0 (5.0, 18.0)	5.0 (3.0, 10.0)	14.0 (8.0, 26.0)	<0.001
**Post-transplant morbidity**				
Any Blood Transfusion	123 (75.0)	40 (63.5)	83 (82.2)	0.01
MI	0 (0.0)	0 (0.0)	0 (0.0)	--
Cardiomyopathy	5 (3.0)	1 (1.6)	4 (3.9)	0.40
Sepsis, 30 days	36 (12.5)	9 (6.9)	27 (17.1)	0.01
Pneumonia, 30 days	42 (14.5)	8 (6.1)	34 (21.5)	<0.001
Abdominal infection, 30 days	26 (9.0)	5 (3.8)	21 (13.3)	0.01
UTI, 30 days	42 (14.5)	11 (8.4)	31 (19.6)	0.01
Positive culture, 30 days	122 (42.2)	34 (26.0)	88 (55.7)	<0.001
Sepsis, 90 days	48 (16.6)	15 (11.5)	33 (20.9)	0.03
Pneumonia, 90 days	57 (19.7)	15 (11.5)	42 (26.6)	0.001
Abdominal infection, 90 days	38 (13.1)	10 (7.6)	28 (17.7)	0.01
UTI, 90 days	69 (23.9)	23 (17.6)	46 (29.1)	0.02
Severe Infection, 90 days	128 (44.3)	44 (33.6)	84 (53.2)	<0.001
Positive culture, 90 days	158 (54.7)	54 (41.2)	104 (65.8)	<0.001

IQR, interquartile-mean range; COD, cause of death; HOC, hepatocellular carcinoma; CCA, cholangiocarcinoma; GVHD, graft versus host disease; PTLD, post-transplant lymphoproliferative disease; LOS, length of hospitalization; Ml, myocardial infarction; UTI, urinary tract infection; PostTx, post-transplant.

**Table 4:** Clinical risk assessment scores for overall population, and stratified by MELD (median, IQR)

Total	Lab MELD <30	Lab MELD ≥ 30		
(N = 289)	(N = 131)	(N = 158)	p-value	
MELD	31.6 (17.4, 38.6)	14.7 (8.8, 22.6)	37.7 (34.2, 41.2)	<0.001
MELD-Na	32.6 (17.5, 38.6)	15.6 (8.8, 24.2)	37.7 (34.4, 41.1)	<0.001
MELD 3.0	32.4 (18.1, 38.3)	16.6 (9.8, 24.8)	37.5 (34.3, 41.1)	<0.001
D-MELD	841.2 (545.6, 1219.9)	543.5 (321.9, 795.8)	1108.9 (836.2, 1496.0)	<0.001
MELD-GRAIL	28.4 (23.1, 30.5)	22.5 (19.8, 25.4)	30.3 (29.1, 31.4)	<0.001
MELD-GRAIL-Na	28.3 (23.2, 30.3)	22.6 (20.0, 25.5)	30.2 (28.9, 31.2)	<0.001
UCLA-FRS	16.5 (10.0, 20.5)	9.5 (4.5, 12.5)	20.3 (18.0, 23.0)	<0.001
BAR	13.0 (6.0, 17.0)	5.0 (3.0, 9.0)	16.0 (14.0, 18.0)	<0.001
SOFT	14.0 (7.0, 25.0)	7.0 (5.0, 13.0)	22.5 (13.0, 29.0)	<0.001
P-SOFT	13.0 (7.0, 26.0)	8.0 (5.0, 11.0)	23.0 (14.0, 28.0)	<0.001
LDRI	1.2 (1.1, 1.5)	1.3 (1.1, 1.6)	1.2 (1.1, 1.4)	0.02
**LIFI**	**33.2 (22.3, 45.9)**	**38.3 (21.6, 48.4)**	**31.3 (23.0, 44.3)**	**0.32**
Low (< 46)	1 (0.3)	0 (0.0)	1 (0.6)	<0.001
Moderate (46–79)	7 (2.4)	0 (0.0)	7 (4.4)	<0.001
High (≥ 80)	116 (40.1)	18 (13.7)	98 (62.0)	<0.001

MELD, Model for End-Stage Liver Disease; GRAIL, GFR assessment in liver disease; UCLA-FRS, University of California Los Angeles Futility Risk Score; BAR, balance of risk; SOFT, survival outcomes following liver transplantation; P-SOFT, predicted survival outcomes following liver transplantation; LDRI liver donor risk index.

**Table 5:** Discrimination power of clinical risk scores compared with LIFI in predicting 1-year post-LT mortality

Risk scores	All patients (N = 289)		MELD < 30 (N = 131)		MELD ≥ 30 (N = 158)	
	C-statistic (95% CI)	p value	C-statistic (95% CI)	p value	C-statistic (95% CI)	p value
**LIFI**	0.85 (0.75, 0.96)	--	0.92 (0.85, 0.98)	--	0.81 (0.63, 0.98)	--
**CCI**	0.62 (0.50, 0.74)	0.01	0.48 (0.32, 0.64)	<0.001	0.69 (0.54, 0.84)	0.30
**SOFT**	0.58 (0.43, 0.73)	0.01	0.50 (0.24, 0.75)	0.002	0.63 (0.48, 0.78)	0.12
**D-MELD**	0.56 (0.44, 0.67)	<0.001	0.65 (0.52, 0.78)	<0.001	0.52 (0.33, 0.70)	0.01
**LDRI**	0.56 (0.45, 0.68)	<0.001	0.63 (0.52, 0.75)	<0.001	0.49 (0.33, 0.66)	0.02
**BAR**	0.56 (0.42, 0.71)	0.003	0.53 (0.31, 0.75)	<0.001	0.59 (0.41, 0.77)	0.06
**MELD**	0.55 (0.40, 0.70)	0.003	0.53 (0.38, 0.68)	<0.001	0.62 (0.42, 0.81)	0.10
**MELD-Na**	0.55 (0.40, 0.70)	0.003	0.52 (0.36, 0.68)	<0.001	0.61 (0.41, 0.81)	0.09
**UCLA-FRS**	0.55 (0.40, 0.69)	0.001	0.52 (0.34, 0.70)	<0.001	0.58 (0.42, 0.75)	0.02
**MELD-GRAIL**	0.54 (0.39, 0.68)	0.001	0.51 (0.35, 0.68)	<0.001	0.56 (0.37, 0.76)	0.048
**Meld GRAIL-Na**	0.54 (0.39, 0.68)	0.001	0.51 (0.34, 0.69)	<0.001	0.57 (0.37, 0.76)	0.049
**MELD 3.0**	0.53 (0.38, 0.67)	0.001	0.54 (0.38, 0.70)	<0.001	0.56 (0.35, 0.76)	0.06

LIFI, Liver Immune Frailty Index; CCI, Charlson Comorbidity Index, age-adjusted; MELD, Model for End-Stage Liver Disease; UCLA-FRS, University of California Los Angeles Futility Risk Score; BAR, Balance of Risk score, SOFT, Survival Outcome Following Liver Transplantation; LDRI, Liver Donor Risk Index

## Data Availability

The deidentified raw data supporting the conclusions of this article will be made available by the authors, without undue reservation upon request.
